# Synergistic theoretical and electrochemical evaluation of sulfonamide-based inhibitors for mild steel corrosion in HCl

**DOI:** 10.1039/d6ra02832b

**Published:** 2026-06-05

**Authors:** Soukaina Alaoui Mrani, Dounia Azzouni, Chahrazad El Abiad, Smail Radi, Fakhreldeen Dabiellil, Mohamed Hussien, Yousef A. Bin Jardan, Muhammad Shahab, Mustapha Taleb

**Affiliations:** a Laboratory of Engineering, Electrochemistry, Modeling and Environment, Faculty of Sciences, University Sidi Mohamed Ben Abdellah Fez Morocco soukaina.alaouimrani@usmba.ac.ma dounia.azzouni@usmba.ac.ma mustaphataleb62@yahoo.fr; b Laboratory of Environment and Applied Chemistry (LCAE), University Mohammed Premier, Faculty of Sciences Oujda Morocco chahra-e@hotmail.fr radismail244@cedocump.space; c University of Bahr el Ghazal Freedowm Stree Wau 91113 South Sudan researcherzem@gmail.com; d Department of Chemistry, Faculty of Science, King Khalid University P. O. Box 9004 Abha 61413 Saudi Arabia mhalmosylhy@kku.edu.sa; e State Key Laboratories of Chemical Resources Engineering, Beijing University of Chemical Technology Beijing 100029 China Shahabkhan1852@gmail.com; f Department of Pharmaceutics, College of Pharmacy, King Saud University P. O. Box 11451 Riyadh Saudi Arabia ybinjardan@ksu.edu.sa

## Abstract

The corrosion inhibition performance of three sulfonamide-based compounds, namely 4-methyl-*N*-(pyridin-2-yl)benzenesulfonamide (SAP4), *N*,*N*′-benzene-1,4-diylbis(4-methylbenzenesulfonamide) (SAP5), and *N*,*N*′-(cyclohexane-1,2-diyl)bis(4-methylbenzenesulfonamide) (SAP6), was investigated for mild steel in 1 M HCl using combined theoretical and experimental approaches. Density functional theory (DFT) calculations provided insight into the electronic properties and reactive sites of the molecules, while Monte Carlo simulations suggested favorable adsorption on the Fe (110) surface. Experimental results from potentiodynamic polarization (PDP) and electrochemical impedance spectroscopy (EIS) demonstrated high inhibition efficiencies, reaching approximately 94%, 95%, and 89% for SAP4, SAP5, and SAP6, respectively, at 1 × 10^−3^ M. The polarization results indicate that the inhibitors affect both anodic and cathodic reactions, suggesting a predominantly mixed-type inhibition behavior. Adsorption studies revealed that the inhibitors follow the Langmuir isotherm model, with high correlation coefficients and negative values of 
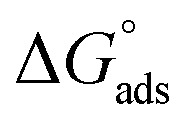
 (−36.66 to −35.32 kJ mol^−1^), indicating spontaneous adsorption involving both physical and chemical interactions. The effect of temperature was evaluated over the range of 298–328 K, where the inhibitors maintained relatively high inhibition efficiencies, although a slight decrease was observed with increasing temperature. Surface analysis further confirmed the formation of a protective adsorbed film on the mild steel surface. Overall, the combined findings highlight the potential of sulfonamide-based compounds, particularly SAP5, as effective corrosion inhibitors for mild steel in acidic environments.

## Abstract 2

Sulfonamide compounds SAP4–SAP6 were evaluated as mild steel corrosion inhibitors in 1 M HCl. Results showed strong adsorption and high inhibition, with SAP5 giving the best efficiency (95%) *via* a stable protective film.

## Graphical abstract

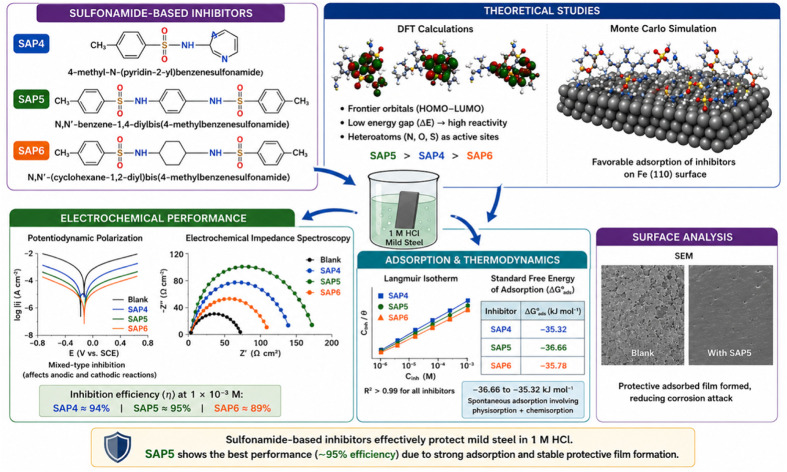

## 1. Introduction

Iron and its alloys, particularly mild steel, remain essential materials in numerous industrial sectors due to their low cost, availability, and favorable mechanical properties.^1,2^ Nevertheless, their exposure to aggressive acidic environments during industrial processes such as pickling and descaling leads to severe corrosion issues, necessitating the development of efficient protection strategies.^3–6^

Among the various mitigation approaches, organic corrosion inhibitors have proven to be highly effective due to their ability to adsorb onto metal surfaces and form protective barriers. Recent studies have highlighted the growing interest in heteroatom-rich organic molecules such as Schiff bases, azoles, ionic liquids, and sulfonamide derivatives, where inhibition efficiency strongly depends on molecular structure, electronic properties, and adsorption behavior.^7–9^

In particular, it has been demonstrated that the presence of electron-donating/accepting groups, π-conjugated systems, and multiple adsorption centers significantly enhances the interaction between inhibitor molecules and metal surfaces.^10^

Similar classes of organic inhibitors incorporating nitrogen, oxygen, and sulfur atoms have been extensively investigated, showing that molecular planarity and extended π-electron delocalization play a crucial role in improving adsorption strength and corrosion inhibition efficiency.^11–15^ In this regard, sulfonamide and tosylamide based compounds have attracted increasing attention due to their ability to form stable adsorbed films through donor–acceptor interactions involving heteroatoms.^16,17^

However, despite these advances, limited studies have systematically explored the effect of structural modulation within tosylamide frameworks on corrosion inhibition performance. It is important to note that the compounds investigated in this study SAP4, SAP5, and SAP6 are structurally derived from classical tosylamide frameworks and therefore are not entirely novel as molecular scaffolds. Nevertheless, the originality of this work lies in the systematic structural modification of these frameworks through the incorporation of different linkers, namely pyridine (SAP4), phenylene (SAP5), and cyclohexane (SAP6). These variations provide a valuable opportunity to investigate the influence of key structural parameters such as π-conjugation, molecular planarity, and steric effects on adsorption behavior and corrosion inhibition performance. In particular, planar and conjugated systems are known to enhance adsorption *via* stronger interaction with metal d-orbitals, whereas non-planar structures may limit surface coverage due to steric hindrance.^18^

To gain deeper insight into these structure–property relationships, computational approaches have become increasingly important in corrosion science. Density functional theory (DFT) is widely used to evaluate electronic properties and identify reactive sites, while Monte Carlo (MC) simulations allow the exploration of adsorption configurations and interaction energies at the metal–solution interface.^19^ In the present work, these complementary methods are applied in a coherent manner to provide a clear molecular level understanding of the inhibition mechanism.

The theoretical findings are further supported by experimental investigations, including potentiodynamic polarization, electrochemical impedance spectroscopy (EIS), and surface characterization techniques. This combined approach enables a comprehensive evaluation of the corrosion inhibition performance of the studied sulfonamide derivatives and provides deeper insight into the relationship between molecular structure and inhibition efficiency in acidic media.

## 2. Materials and methods

### 2.1. Specimens and materials

Mild steel substrate has the chemical composition of (wt%) 99.30 Fe, 0.21 C, 0.38 Si, 0.09 P, 0.05 S, 0.01 Mn and 0.01 Al. To be ready for the experiment, the specimens were abraded with SiC paper (100–1800 grit), degreased with acetone, washed using distilled water and then air-dried. The surface preparation procedure was carefully controlled to ensure reproducibility, and the same surface condition was systematically used for all electrochemical measurements, including potentiodynamic polarization (PDP) and electrochemical impedance spectroscopy (EIS).

The acid environment was obtained by diluting 37% hydrochloric acid (HCl, Fisher Scientific) with distilled H_2_O until a 1 M HCl was obtained.

Tosyl chloride, 2-aminopyridine, benzene-1,4-diamine, (1*S*,2*S*)-1,2-diaminocyclohexane was received from Merck.

Dichloromethane (CH_2_Cl_2_), tetrahydrofuran (THF), potassium carbonate (K_2_CO_3_), sodium carbonate (Na_2_CO_3_), sodium sulfate (Na_2_SO_4_) was obtained from Alfa Aesar and used without further purification.

### 2.2. Synthesis of inhibitor molecules

#### 2.2.1. Synthesis of 4-methyl-*N*-(pyridin-2-yl)benzenesulfonamide (SAP4)

4-methyl-*N*-(pyridin-2-yl)benzenesulfonamide (SAP4) was synthesized by reacting tosyl chloride (1) and 2-aminopyridine (2) dissolved in CH_2_Cl_2_ taking 1 : 1 ratio. To the mixture, a diluted solution of K_2_CO_3_ was added until the pH approaches 10 and the resulting white compound was collected after filtration of the organic phase and dried under vacuum.^20,21^

#### 2.2.2. Synthesis of *N*,*N*′-(1,4-phenylene)bis(4-methylbenzenesulfonamide) (SAP5)

In order to synthesize *N*,*N*′-(1,4-phenylene)bis(4-methylbenzenesulfonamide) (SAP5), benzene-1,4-diamine (3) (2.701 g, 25 mmol) was dissolved in 100 ml CH_2_Cl_2_ at room temperature and one equivalent of Na_2_CO_3_ was added. Two equivalents of chloride solution of *p*-toluene sulfonyl (1) (9.4995 g, 50 mmol) were taken in CH_2_Cl_2_ and added subsequently. The reaction mixture was stirred at room temperature for two days and then filtered to remove sodium bicarbonate and sodium chloride. The filtrate was collected and stored.^20,21^

#### 2.2.3. Synthesis of *N*,*N*′-(cyclohexane-1,2-diyl)bis(4-methylbenzenesulfonamide) (SAP6)

**Fig. 1:**
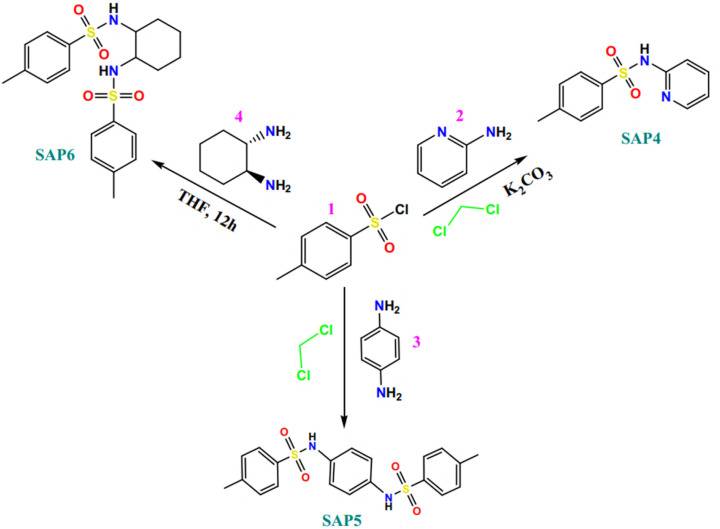
Synthesis route and structures of sulphonamide-based corrosion inhibitors, SAP4, SAP5 and SAP6.

A mixture of (1*S*,2*S*)-1,2-diaminocyclohexane solution (4) (2.854 g, 25 mmol) in THF (100 ml) was cooled to 0 °C and a solution of *p*-toluenesulfonyl chloride (1) (4.749 g, 50 mmol) in THF (10 ml) were added dropwise over 0.5 to 1 h. After its complete addition, the mixture was then allowed to warm up to room temperature and stirred for 12 h. After that, the solvent was removed under reduced pressure to get a crude product. The obtained crude product was resolved in CH_2_Cl_2_ and washed by Na_2_CO_3_. The aqueous solution was then extracted with 30 ml of CH_2_Cl_2_. The combined layer was dried (anhydrous Na_2_SO_4_) and filtered to obtain the product: *N*,*N*′-(1*S*,2*S*)-cyclohexane-1,2-diylbis(4-methylbenzenesulfonamide).^20,21^ The synthesis route and structures of the studied corrosion inhibitors are displayed in Fig. 1.

### 2.3. Theoretical simulation

Density functional theory (DFT) was performed using Gaussian 09 software with B3LYP functional along with 6-31G (d,p) basis set to optimize the geometry of corrosion inhibitor molecules. The optimized geometries were confirmed to correspond to true minima by the absence of imaginary frequencies. The electron density distribution in the optimized geometry of corrosion inhibitors, as well as their relevant DFT descriptors derived from frontier molecular orbitals, were computed in the aqueous phase with water as a solution. Furthermore, to simulate molecules adsorption on the iron surface and to determine the associated adsorption energy parameters, the Monte Carlo (MC) simulation technique was implemented. For this purpose, an initial optimization of the geometry of the corrosion inhibitors was performed by ‘Forcite’ module (using COMPASS force field) *via* Materials Studio™ Software (7.0 version). The Fe (110) crystals with edges of 50 Å were constructed, and then enlarged to create an 11 × 11 super cells and a vacuum cell was created, so that the created simulation box can achieve the most stable inhibitor adsorption on iron surface. Then each of the optimized corrosion inhibitor was loaded in the created Fe (110) simulation box along with the addition of 200H_2_O molecules using simulated annealing technique in ‘Adsorption Locator’ module in the Materials Studio™ Software (version 7.0) to obtain the adsorption of the corrosion inhibitor with minimum energy. The reported adsorption energies correspond to the total energy of the simulation system and are expressed in kcal mol^−1^. Negative values of adsorption energy indicate a spontaneous and stable adsorption process.

### 2.4. Weight loss measurements

Weight loss measurement was performed by immersing the metal specimens in vials containing the 1 M HCl in the absence and the presence of different concentrations of inhibitors for 6 h duration at a temperature of 298 K (the samples are maintained in a thermostatic bath). Afterwards, the samples were carefully taken out from the vials, rinsed with distilled water, and degreased with acetone, dried, and then weighed for the determination of the mass loss. All experiments were carried out in triplicate, and the reported values correspond to the mean ± standard deviation.

### 2.5. Electrochemical measurements

The electrochemical studies, such as potentiodynamic polarization (PDP) and electrochemical impedance spectroscopy (EIS) were conducted under static (unstirred) conditions in order to avoid hydrodynamic effects that could influence the adsorption behavior of inhibitors and the electrochemical response and controlled temperature (using a thermostatic bath). All measurements were performed in triplicate, and the reported electrochemical parameters correspond to the mean values obtained from three independent experiments, with uncertainties expressed as standard deviations. The raw data were obtained using a VersaSTAT 4 potentiostat/galvanostat and processed using VersaStudio software. Both PDP and EIS tests were performed utilizing a “conventional three-electrode cell” system equipped with Ag/AgCl as reference electrode, an auxiliary platinum electrode and mild steel specimens as a working electrode with exposed surface area of 1 cm^2^. Before each experiment, the open circuit potential (OCP) was held for 30 minutes until stabilization. The PDP measurements were performed by polarizing the samples from ±250 mV with a scan rate of 0.5 mV s^−1^. The EIS measurements were carried out in the frequency range of 100 kHz to 10 mHz using a sinusoidal AC perturbation amplitude of 10 mV at the OCP.

### 2.6. Surface analysis

Prior to surface analysis, the metal specimens were polished, rinsed, and dried properly. These specimens were then dipped into HCl (1 M) for 6 h in both uninhibited and inhibited systems using an optimum concentration of the inhibitor. A scanning electron microscope (SEM) FEI Quanta 200, was used to analyze the sample surfaces.

## 3. Results and discussion

### 3.1. Quantum chemical calculations

**Fig. 2:**
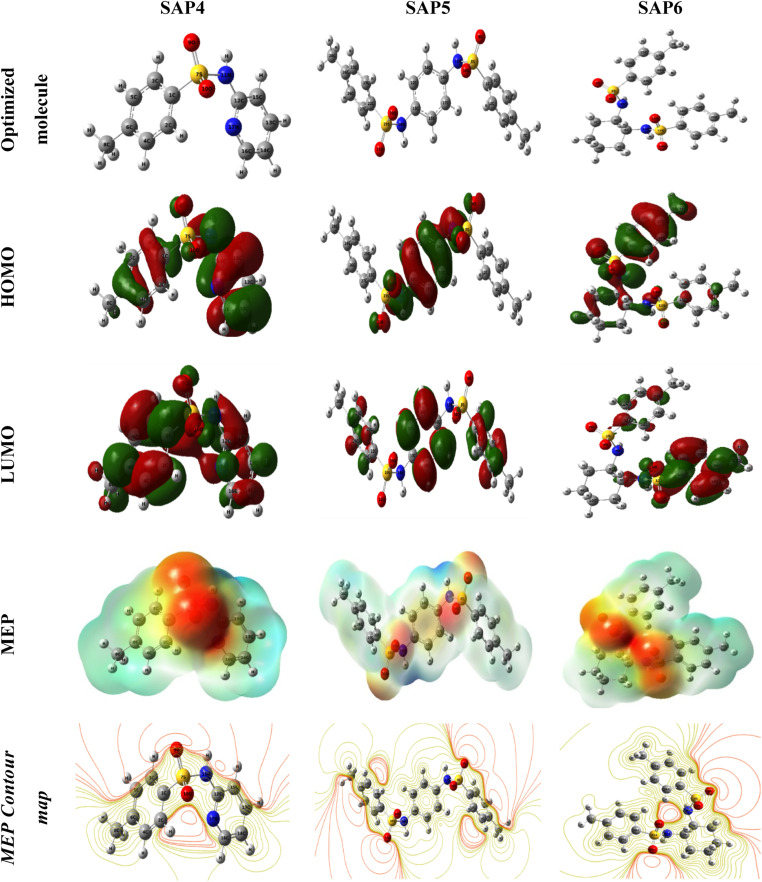
Optimized geometrical configurations, electronic distribution in HOMO and LUMO, MEP plots and MEP contour mapping of the corrosion inhibitors (SAP4, SAP5 and SAP6).

DFT calculations constitute a powerful tool for understanding the relationship between molecular structure and corrosion inhibition performance. It is commonly utilized to study correlation between the molecular structure of inhibitors and their corrosion inhibition capacity. Molecular arrangement in space and electronic distribution on the frontier molecular orbitals (highest occupied molecular orbital, HOMO, and lowest unoccupied molecular orbital, LUMO) are the main factors that helps in predicting the interaction and adsorption capacity of molecules. The optimized geometrical configurations, electronic distribution in the frontier molecular orbitals (HOMO and LUMO), molecular electrostatic potential (MEP) and MEP contour map of the studied molecules (SAP4, SAP5 and SAP6) are shown in Fig. 2. The HOMO of corrosion inhibitor is capable of donating electrons to the vacant d-orbital of the metal leading to the formation of a coordination bond. On the other hand, the LUMO of corrosion inhibitors are capable to accommodate the back donated electrons from the metal surface.^22^ It has been observed from Fig. 2 that the electron distribution on the HOMO and LUMO of the sulfonamide-based corrosion inhibitors is distributed along the most reactive sites of the molecular skeleton. The possession of electron rich and deficient sites within the corrosion inhibitor molecules suggest that these molecules can donate their electron to the electron deficient metal d-orbitals and capable to accommodate the excess electrons reverting from metal centers towards antibonding orbitals of corrosion inhibitors. A careful observation of Fig. 2 shows that favorable electron donating sites (HOMO) are mostly situated around the S, O and N heteroatoms of the molecules, whereas electron accepting sites (LUMO) are localized in pyridine, benzene regions.^23^ Hence, it can be said that the electron donations form S, O and N heteroatoms of molecules facilitates the adsorption of sulfonamide molecules counter. Additionally, electron accepting capability of pyridine moiety (in case of SAP4), while benzene moieties (in SAP5 and SAP6) enhances the studied inhibitor adsorption on the metal surface. Thus, synergistic electron donation as well as acceptance facilitates the strong adsorption of the designed sulfonamide inhibitors on the targeted mild steel surfaces.

Inhibitor molecules adsorption on metal surface is also related to energies of highest occupied molecular orbital *i.e.*, *E*_HOMO_ and lowest unoccupied molecular orbital *i.e.*, *E*_LUMO_. The higher *E*_HOMO_, better will be the molecule electron donation capacity towards the metal center. While, the lower *E*_LUMO_, the greater will be the molecule electron acceptance capacity. The greater feasibility of electron sharing through donation and acceptance of electrons leads to a strong inhibitor's adsorption on metal surface atoms, facilitates a thin protective film formation.

The stronger and more impermeable is the adherent film, the greater the corrosion inhibition effectiveness. The obtained *E*_HOMO_ and *E*_LUMO_ values of the studied sulfonamide inhibitors (SAP4, SAP5 and SAP6) have been tabulated in Table 1. We note that *E*_HOMO_ values remarkably increased in the order SAP6 < SAP4 < SAP5, which indicates that electron donating capacity order is SAP5 > SAP4 > SAP6. It implies that SAP5 easily donates electrons to the vacant d-orbital of metal surface atoms in compared to SAP4 and SAP6. Whereas, *E*_LUMO_ values illustrated that the electron acceptance capability decreases in the sequence of SAP4 ≈ SAP5 > SAP6. Another important parameter that is also important to elucidate chemical reactivity of the inhibitor molecule is the energy gap (Δ*E*). When Δ*E* decreases, chemical reactivity of inhibitor rises, which reflect an increase in the molecules adsorption onto metal surface.^24^Table 1, suggest Δ*E* values decreases in the following order: SAP6 > SAP4 > SAP5. These results align well with the experimental findings. In addition, the Molecular Electrostatic Potential (MEP) analysis was performed to identify the active sites of the studied molecules. As reported in the literature, areas with high electron density appear in red or orange, whereas regions with low electron density are shown in blue.^25,26^Fig. 2 reveals, in MEP high electron density is mainly localized on the sulfonyl group, <svg xmlns="http://www.w3.org/2000/svg" version="1.0" width="10.400000pt" height="16.000000pt" viewBox="0 0 10.400000 16.000000" preserveAspectRatio="xMidYMid meet"><metadata> Created by potrace 1.16, written by Peter Selinger 2001-2019 </metadata><g transform="translate(1.000000,15.000000) scale(0.011667,-0.011667)" fill="currentColor" stroke="none"><path d="M80 1160 l0 -40 40 0 40 0 0 -40 0 -40 40 0 40 0 0 -40 0 -40 40 0 40 0 0 -40 0 -40 40 0 40 0 0 -40 0 -40 40 0 40 0 0 -40 0 -40 40 0 40 0 0 -40 0 -40 40 0 40 0 0 80 0 80 -40 0 -40 0 0 40 0 40 -40 0 -40 0 0 40 0 40 -40 0 -40 0 0 40 0 40 -40 0 -40 0 0 40 0 40 -40 0 -40 0 0 40 0 40 -80 0 -80 0 0 -40z M560 520 l0 -40 -40 0 -40 0 0 -40 0 -40 -40 0 -40 0 0 -40 0 -40 -40 0 -40 0 0 -40 0 -40 -40 0 -40 0 0 -40 0 -40 -40 0 -40 0 0 -40 0 -40 -40 0 -40 0 0 -40 0 -40 80 0 80 0 0 40 0 40 40 0 40 0 0 40 0 40 40 0 40 0 0 40 0 40 40 0 40 0 0 40 0 40 40 0 40 0 0 40 0 40 40 0 40 0 0 80 0 80 -40 0 -40 0 0 -40z"/></g></svg> S(=O)_2_. It reflects that these regions of the molecules are the most reactive centers that possess immense ability to facilitate the adsorption on metal surfaces. Herein, it can be said that the incorporation of S(=O)_2_ groups in the molecular structure of sulfonamide-based inhibitors contribute favorably to their adsorption behavior on metal surfaces. Additionally, as observed in the MEP contour map, the red contours (indicative of electron-rich regions) are predominantly concentrated around the heteroatoms in sulfonamide molecules, which is consistent with the electron density distribution in the HOMO.

**Table 1:** Quantum chemical parameters of the neutral form of corrosion inhibitors (SAP4, SAP5 and SAP6)

Parameters	SAP4	SAP5	SAP6
*E*_HOMO_ (eV)	−6.4664	−6.1475	−6.8618
*E*_LUMO_ (eV)	−1.1108	−1.0988	−1.0362
Δ*E* (eV)	5.3556	5.0486	5.8255
*χ* (eV)	3.7886	3.6231	3.9490
*γ* (eV)	2.6778	2.5243	2.9128
*σ* (eV^−1^)	0.3734	0.3961	0.3433
Δ*N*	0.1926	0.2371	0.1495

Furthermore, to assess the potential of the investigated sulfonamide derivatives to engage in electron transfer with metal surface atoms, the fraction of electrons transferred (Δ*N*) was calculated. According to Koopmans' theorem, key molecular descriptors including ionization potential (*I*), electron affinity (*A*), electronegativity (*χ*), and global hardness (*γ*) were determined for the inhibitors.^23,24^ These parameters are interrelated through the following equations eqn (1)–(4):

1 *I* = −*E*_HOMO_

2 *A* = −*E*_LUMO_

3
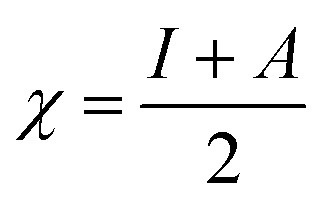
4
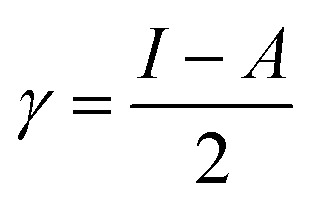


By considering aforesaid values, Δ*N* was calculated using eqn (5):5
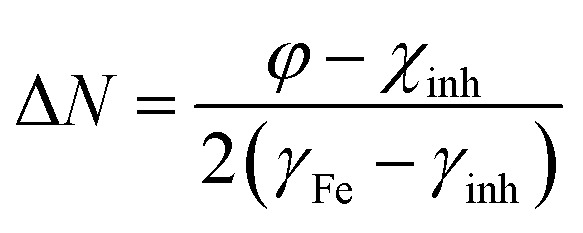
where, *χ*_inh_ is the inhibitor's electronegativity, *γ*_Fe_ and *γ*_inh_ are the hardness of iron and inhibitor molecules, respectively. The considered value *γ*_Fe_ = 0 eV and *φ* for Fe (110) surface is 4.82 eV. The Fe (110) crystallographic surface was chosen because of its favorable stability and closely packed nature, which make it representative of realistic metallic surfaces.^27^

It is considered that when the Δ*N* value is greater than zero, electron transfer is assumed to occur from the inhibitor molecules to the metal surface atoms.^28^ Furthermore, several researches pointed out that there is an enhancement of the electron-donating capacity of molecules when the Δ*N* < 3.6.^29^ It can be seen in Table 1 that all calculated Δ*N* values for the studied corrosion inhibitors are positive and less than 3.6. Therefore, all the sulfonamide-based molecules are capable of donating electrons to the metal surface atoms and thereby facilitate adsorption on the metal surfaces. It has also been found that Δ*N* values follow the order SAP5 > SAP4 > SAP6, which is corroborated well with the experimental findings.

In addition to the characteristic properties listed above, global softness (*σ*, reciprocal of hardness) is closely related to the chemical reactivity of a molecule. According to the Hard and Soft Acids and Bases (HSAB) principle, metals act as soft acids while inhibitor molecules behave as soft bases.^4,23^ Consequently, adsorption is favored for inhibitors exhibiting higher softness values. As shown in Table 1, the softness values follow the order SAP5 > SAP4 > SAP6, which correlates with the expected sequence of inhibition efficiency. Therefore, the experimental results fully corroborate the theoretical predictions.

### 3.2. Monte Carlo simulation

**Fig. 3:**
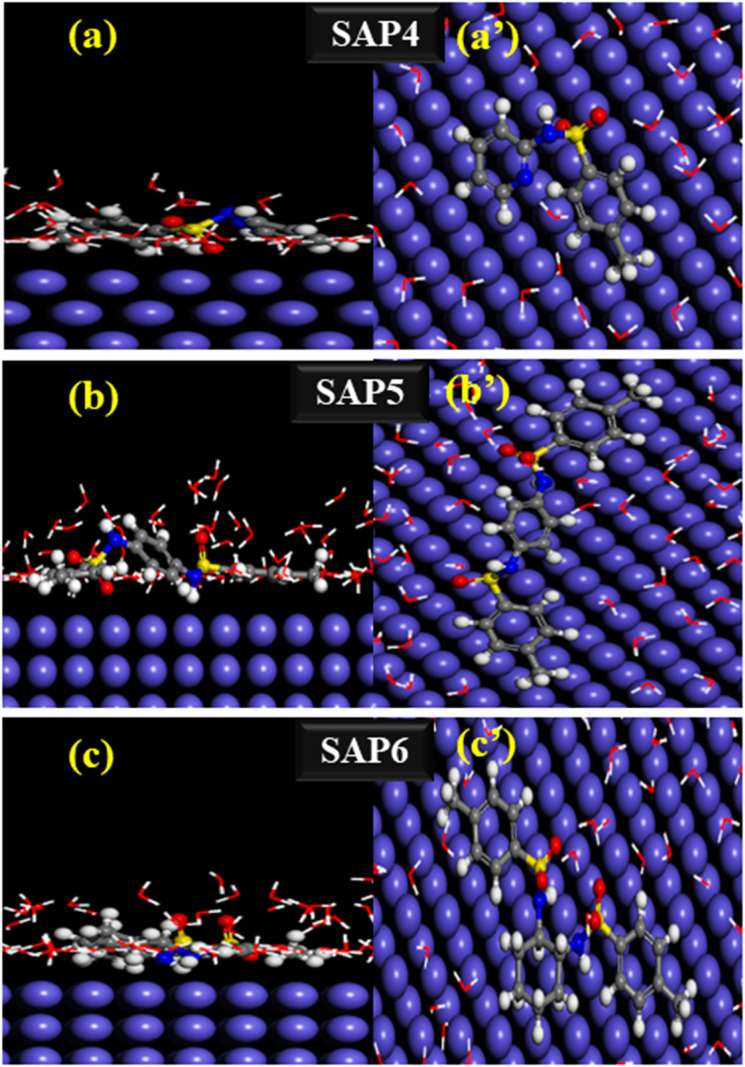
Equilibrium adsorption configuration corrosion inhibitors (a and a′) SAP4, (b and b′) SAP5 and (c and c′) SAP6 on Fe (110) surface [a–c refers to the side view, and a′–c′ refers to the top view].

MC simulations were conducted to examine the adsorption behavior of organic molecules on the Fe (110) surface within an aqueous phase.^30,31^ The results obtained from the simulated adsorption of sulfonamide-based molecules (herein, SAP4, SAP5 and SAP6) using MC simulation approach are presented in Fig. 10, with the corresponding energy parameters summarized in Table 2. Fig. 3(a–c) and (a′–c′) display the side and top views, respectively, of the most stable, lowest adsorption energy configurations of SAP4, SAP5, and SAP6 on the Fe (110) surface.

**Table 2:** Energy parameters of corrosion inhibitors (SAP4, SAP5 and SAP6) adsorbed on Fe (110) surface obtained from MC simulation

System	*E*_ads_ (kcal mol^−1^)
Fe(110)/SAP4/200H_2_O	−2968.51
Fe(110)/SAP5/200H_2_O	−3037.67
Fe(110)/SAP6/200H_2_O	−2941.69

It is observed from Fig. 3 that inhibitor molecules designated as SAP4 and SAP5 adsorbed onto Fe (110) surface in an almost parallel manner. Whereas, in SAP6, 4-methylbenzenesulfonamido segment has been adsorbed and rest of the part are not adsorbed probably due to steric effect. The adsorption of sulfonamide derivatives occurs through donor–acceptor (D–A) interactions between reactive atoms (heteroatoms) in the inhibitor molecules and the metal surface atoms. This interaction leads to the formation of a protective thin film that shields the metal from aggressive corrosive species, thereby inhibiting the corrosion process.

Furthermore, the energy parameters associated with the adsorption of corrosion inhibitors onto the metal surface were analyzed. It is well established that a higher magnitude of negative adsorption energy corresponds to stronger and more stable adsorption. Additionally, the negative values of the adsorption energies indicate that the adsorption process occurs spontaneously on the targeted metal surface atoms.^32–35^ From *E*_ads_ values (*vide*Table 2), it is evident that adsorption energy follows the order of SAP5 > SAP4 > SAP6. It suggests that SAP5 adsorb on the metal surfaces more prominently compared to SAP4 and SAP6. Stronger adsorption facilitates the formation of a more compact and stable protective film on the metal surface. Therefore, it can be concluded that the Monte Carlo simulation results are in good agreement with both the quantum chemical calculations and the experimental findings.

### 3.3. Mechanism of adsorption and corrosion inhibition

**Fig. 4:**
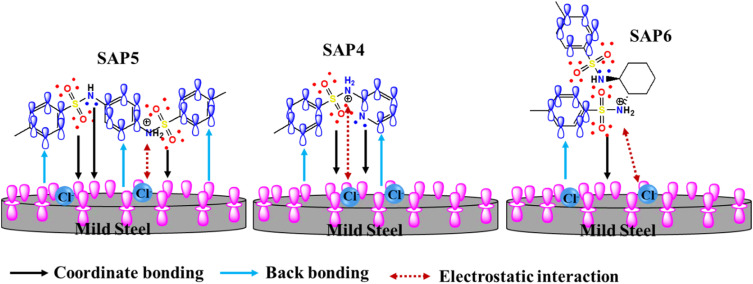
Adsorption mechanism of sulphonamide-based corrosion inhibitors (SAP4, SAP5 and SAP6) on mild steel.

It can be seen from the geometrical structure of presently explored sulfonamide-based corrosion inhibitors that the SAP5 comprised of two 4-methylbenzenesulfonamido units connected by a benzene ring at *p*-position; while SAP6 comprised of two 4-methylbenzenesulfonamido units connected by a cyclohexane ring at *o*-position. In addition, SAP4 consists of one 4-methylbenzenesulfonamido unit connected to the pyridine unit (*vide*Fig. 1). Considering the geometrical orientation, it is obvious that the SAP4 molecule may exist in a planar orientation. The SAP5 molecule may also exist in planar orientation and the delocalization of π-electron might take place. On the other hand, SAP6 molecule will be unable to orient itself in the planar configuration due to the presence of steric hindrance of the two bulky 4-methylbenzenesulfonamido units; connected by a cyclohexane ring at *o*-position (*vide*Fig. 4). Herein, the corrosion inhibition trends can be attributed to the presence of heteroatoms bearing lone pairs of electrons, extended π-bond conjugation or resonance effect and planarity of the molecule.^36–38^ Lone pairs of electrons on heteroatoms like nitrogen, oxygen, and sulfur, together with delocalized π-electrons, coordinate with the vacant d-orbitals of metal surface atoms, promoting adsorption.^39–43^ Molecules containing a higher number of heteroatoms and delocalized π-electrons exhibit stronger adsorption on the metal surface.^44,45^ Thus, it can be said that SAP5 having greater number of heteroatoms and delocalized π-electrons throughout its structure, facilitates its adsorption onto metal surfaces and cover a larger surface area than molecules SAP6 and SAP4. In contrast, if comparison has been made between SAP4 and SAP6, the geometrical configuration of SAP6 does not allow the entire molecule to be aligned in a planar orientation, which hinders the approach of heteroatoms and π-electrons in the close approximate of metal surface atoms. This minimizes SAP6 adsorption capability compared to SAP4 (*vide*Fig. 4). The adsorption ability of the molecules *via* chemical as well as physical adsorption has been presented schematically in Fig. 4.

The corrosion inhibition mechanism of sulfonamide-based compounds involves both physical and chemical adsorption on the mild steel surface. In acidic media, the metal surface is positively charged; however, chloride ions (Cl^−^) from HCl adsorb first, creating a negatively charged layer that facilitates electrostatic interaction with protonated inhibitor molecules (physisorption).

In addition, chemisorption occurs through donor–acceptor interactions between lone pair electrons on heteroatoms (N, O, S) and vacant d-orbitals of Fe atoms. π-electrons from aromatic rings further enhance adsorption *via* π–d interactions.

Moreover, the formation of Fe–inhibitor complexes and possible interaction with corrosion products contributes to the formation of a compact protective film. This adsorbed layer acts as a barrier, reducing both anodic dissolution and cathodic hydrogen evolution reactions.

The proposed adsorption mechanism for sulfonamide-based molecules on mild steel surface has clearly been evidenced by MD simulation results. The simulated adsorption of presently explored sulfonamide-based corrosion inhibitors also revealed that the adsorption energies in the order SAP5 > SAP4 > SAP6. The equilibrium adsorption configuration also revealed that SAP5 adsorbs well in comparison to the SAP6 (*vide*Fig. 3).

### 3.4. Weight loss measurements

The weight loss measurements of the test specimens were recorded both before and after immersing them for 6 hours in 1 M HCl solution with varying concentrations (0 to 1 × 10^−3^ M) of corrosion inhibitor were performed at room temperature (298 K). The corrosion rate (*ν*) and percentage inhibition efficiencies (IE_*ν*_%) of the three inhibitors in a 1 M HCl medium was determined by eqn (6) and (7), respectively:6
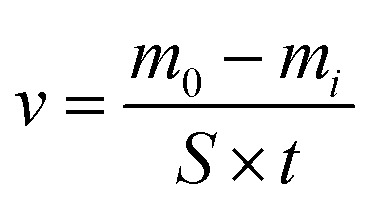
7
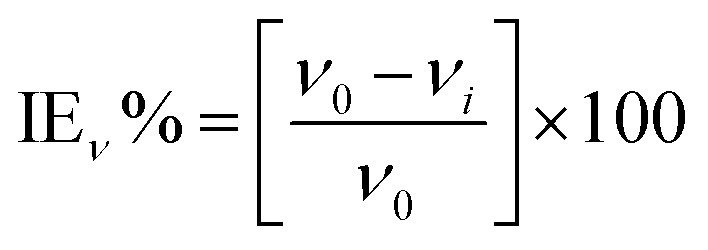
where, *m*_0_ and *m*_*i*_ represent the masses of the samples before and after immersion in the test solutions; *S* and *t* represent the surface area of substrate and time of exposure; *ν*_*i*_ and *ν*_0_ represent the corrosion rates of mild steel in the presence and absence of corrosion inhibitors, respectively. The calculated values of *ν* and IE_*ν*_% are presented in Table 3.

**Table 3:** Calculated corrosion rate (*ν*) and corrosion inhibition efficiencies (IE_*ν*_%) of studied corrosion inhibitors with variation of concentration determined from weight loss measurement

Medium	Concentration (M)	Corrosion rate (mg cm^−2^ h^−1^)	Inhibition efficiency IE_*v*_%
HCl	1	0.6532	—
SAP4	1 × 10^−3^	0.0522	92
	5 × 10^−4^	0.1104	83
	1 × 10^−4^	0.1829	72
	5 × 10^−5^	0.1959	70
SAP5	1 × 10^−3^	0.0718	89
	5 × 10^−4^	0.1175	82
	1 × 10^−4^	0.1437	78
	5 × 10^−5^	0.2547	61
SAP6	1 × 10^−3^	0.0914	86
	5 × 10^−4^	0.1633	75
	1 × 10^−4^	0.2025	69
	5 × 10^−5^	0.3135	52

It appears from the tabulated data in Table 3 that the corrosion rate of mild steel decreases after the addition of corrosion inhibitors into corrosive HCl solution. Furthermore, it has also been found that the corrosion rate decreases upon increase of the concentration of added sulfonamide compounds. These results confirm the effective adsorption of the inhibitors on the mild steel surface, leading to significant corrosion mitigation.^46^ It has been found that maximum inhibition efficacy attain was around 90% at the concentration of 1 × 10^−3^ M and it follows the trend of SAP4 ≈ SAP5 > SAP6. It might be said that the sulfonamide derivatives adsorbed well on the targeted mild steel surface and form a strongly adsorbed protective layer which is efficient in of the mild steel corrosion inhibition.

### 3.5. Potentiodynamic polarization measurements

**Fig. 5:**
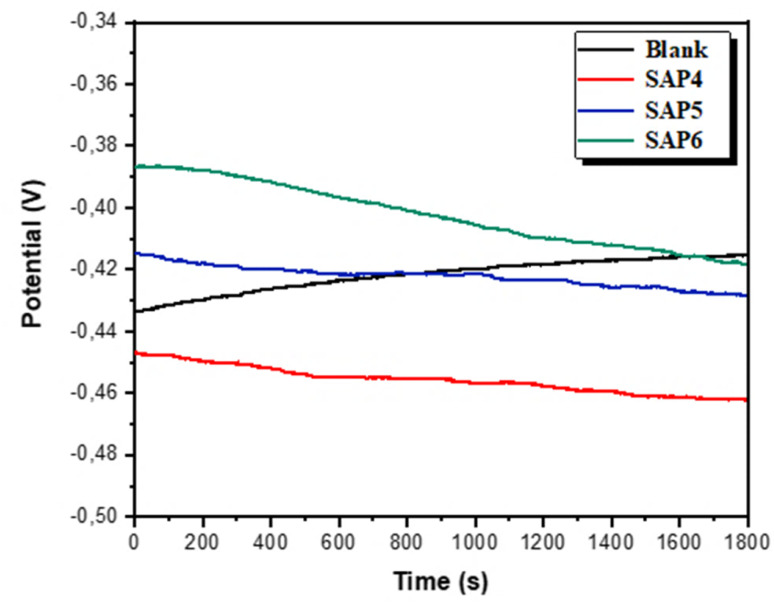
Open circuit potential (OCP) curves for mild steel immersed in 1 M HCl solution, in the absence and presence of the highest concentration of corrosion inhibitors.

Prior to the electrochemical measurements, it is necessary to monitor OCP of the system to unveil the stability of electrolytic system.^47^ In the present experiment, the OCP plot represented by the potential *vs.* time *vide*Fig. 5 was recorded for mild steel samples immersed in 1 M HCl containing optimum concentrations of each of the three inhibitors (SAP4, SAP5 and SAP6) for 30 min duration. The obtained OCP curved ascertained the attainment of the steady state of the electrochemical system under investigation.

**Fig. 6:**
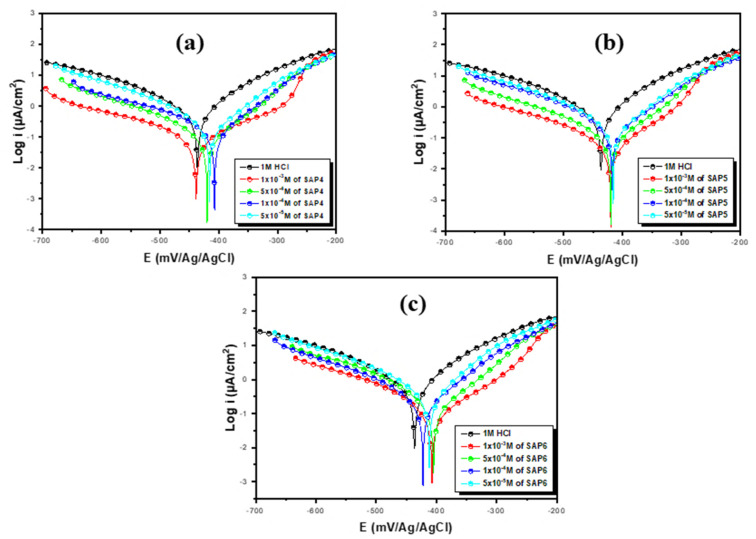
PDP Tafel plots obtained for mild steel with and without different concentrations of sulphonamide-based corrosion inhibitors, (a) SAP4, (b) SAP5 and (c) SAP6 in 1 M HCl at 298 K.

After the stabilization of the open circuit potential (OCP), all electrochemical measurements were conducted. The potentiodynamic polarization (PDP) curves for mild steel immersed in 1 M HCl, both in the absence and presence of various concentrations of sulfonamide-based corrosion inhibitors at 298 K, are shown in Fig. 6. Electrochemical parameters, including corrosion current density (*i*_corr_), cathodic Tafel slope (*β*_c_) and corrosion potential (*E*_corr_) extracted from the Tafel plots. The corresponding corrosion inhibition efficiency (*η*_PP_%) was also determined using eqn (8) are shown in Table 4.8
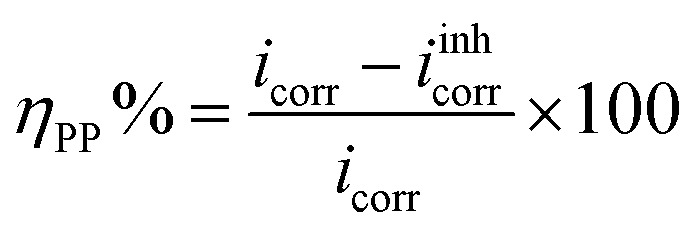
where, *i*_corr_ and *i*^inh^_corr_ represent the corrosion current densities in the absence and presence of the corrosion inhibitor, respectively.

**Table 4:** Electrochemical parameters derived from potentiodynamic polarization curves obtained in the absence and presence of various concentrations of corrosion inhibitors (SAP4, SAP5 and SAP6)

Medium	Concentration (M)	−*E*_corr_ (mV/Ag/AgCl)	*i*_corr_ (µA cm^−2^)	−*β*_c_ (mV dec^−1^)	*η*_PP_%
Blank	1	437 ± 1.5	983 ± 2.8	150 ± 3.0	—
SAP4	1 × 10^−3^	439 ± 2.0	58 ± 2.4	102 ± 2.7	94
	5 × 10^−4^	420 ± 1.7	120 ± 1.6	130 ± 1.1	87
	1 × 10^−4^	408 ± 2.3	173 ± 1.1	138 ± 2.9	82
	5 × 10^−5^	416 ± 1.4	354 ± 3.2	139 ± 2.4	64
SAP5	1 × 10^−3^	420 ± 2.1	50 ± 1.4	110 ± 2.3	95
	5 × 10^−4^	419 ± 2.5	120 ± 2.2	121 ± 2.7	88
	1 × 10^−4^	417 ± 1.9	274 ± 1.5	128 ± 3.3	72
	5 × 10^−5^	416 ± 1.7	310 ± 2.5	130 ± 1.3	68
SAP6	1 × 10^−3^	407 ± 1.1	112 ± 1.9	111 ± 1.8	89
	5 × 10^−4^	406 ± 1.4	202 ± 2.1	112 ± 2.4	79
	1 × 10^−4^	423 ± 2.2	290 ± 1.9	135 ± 1.5	71
	5 × 10^−5^	412 ± 2.9	457 ± 2.0	133 ± 1.1	54

The polarization curves analysis indicate that both anodic (metal dissolution) and cathodic (hydrogen evolution) reactions are affected by the addition of inhibitors to the corrosive medium (*vide*Fig. 6). Although, it can be observed that the shifting of anodic curves is more pronounced compared to that of the cathodic plots upon addition of sulfonamide-based corrosion inhibitors. These findings reflect that the anodic reaction of mild steel was inhibited by the addition of sulfonamide-based corrosion inhibitors.^48^ Additionally, anodic, and cathodic Tafel slopes vary slightly which is independent of the concentrations of inhibitors. It suggests that the oxidation and reduction mechanism in the aggressive solutions before and after adding the inhibitors undergo a similar pattern. Hence, it can be inferred that the studied sulfonamide derivatives functioned as corrosion inhibitors by spontaneously adsorbing onto the metal surface, thereby blocking the active corrosion sites.^49^ Furthermore, the obtained electrochemical parameters are tabulated in Table 4 reveals that *i*_corr_ values of the working electrode decrease with the increase in inhibitors concentrations which suggest the associated increase in the corrosion inhibition efficiencies. It has been found that the application of an optimum concentration of corrosion inhibitors (1 × 10^−3^ M) resulted in the corrosion inhibition efficiencies trends in the order: SAP5 > SAP4 > SAP6.

### 3.6. Electrochemical impedance spectroscopy measurements

**Fig. 7:**
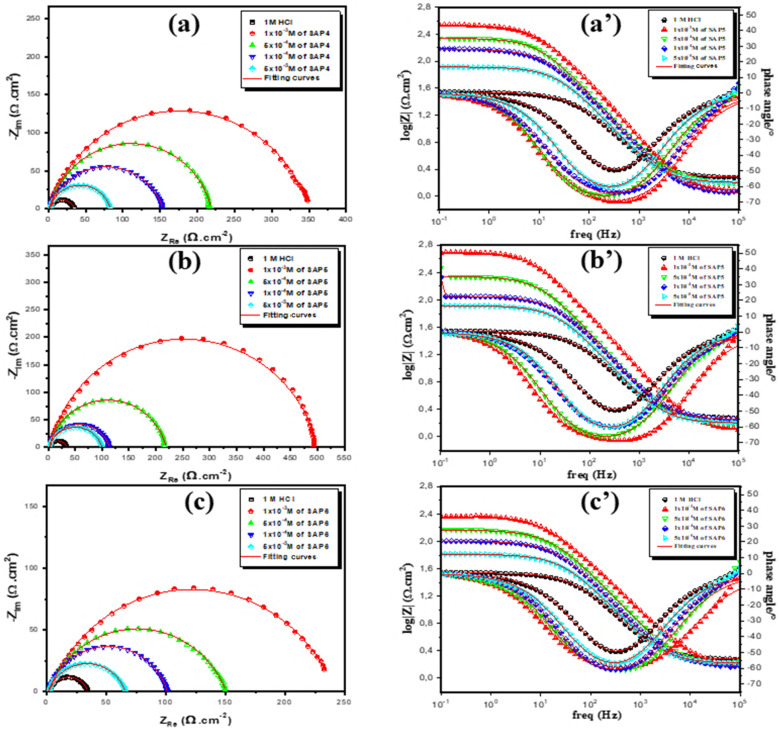
EIS Nyquist and Bode plots of mild steel obtained for corrosion inhibitors, (a and a′) SAP4, (b and b′) SAP5 and (c and c′) SAP6 in 1 M HCl at 298 K.

Electrochemical impedance spectroscopy (EIS) is a non-invasive technique which has also been utilized to explore and understand the physicochemical properties during corrosion as well as its inhibition mechanism in the solution state. The present study utilizes EIS to elucidate the mechanism by which the synthesized corrosion inhibitors impede the corrosion process. The EIS results are presented as Nyquist and Bode plots in Fig. 7. The obtained plots were acquired before and after adding various inhibitors concentrations in 1 M HCl solution. It has been found in the present investigation that obtained the Nyquist plot exhibits a single semicircle, and the Bode plot indicates one distinct time constant. This behavior clearly suggests that, in the absence of inhibitors, the corrosion of mild steel is predominantly controlled by charge transfer processes. It is well established that the difference between the high- and low-frequency regions in the Nyquist plot corresponds to the charge transfer resistance (*R*_ct_). Moreover, *R*_ct_ is associated with metal and outer Helmholtz plane, and it must be in consistent with the developed resistance.^7,50–52^ Additionally, resistance can arise from species accumulation, referred to as accumulation resistance (*R*_a_), and from mass transport limitations, known as diffuse layer resistance (*R*_d_). All these resistances contribute to the overall corrosion process and must be taken into account. Consequently, the polarization resistance (*R*_p_) is defined as the difference between the real impedance values at high and low frequencies.^7,51^

Furthermore, the addition of organic corrosion inhibitors at different concentrations to the corrosive medium results in a noticeable increase in the Nyquist plot semicircle diameter, indicating greater adsorption of inhibitor molecules on the metal surface. Therefore, it is appropriate to include a film resistance (*R*_f_) component within the overall polarization resistance (*R*_p_). Consequently, when corrosion inhibitors are introduced into the corrosive medium, *R*_p_ represents the combined effect of all relevant resistances, namely charge transfer resistance (*R*_ct_), diffuse layer resistance (*R*_d_), accumulation resistance (*R*_a_), and film resistance (*R*_f_).^7,51,53^

Moreover, the Nyquist plot provides valuable insights into the capacitive behavior of both inhibited and uninhibited metal surfaces. In this study, the Nyquist plots deviate from ideal semicircular shapes and instead appear depressed towards the real axis, indicating non-ideal capacitive behavior. This distortion is commonly attributed to surface heterogeneity and microscopic roughness present on the electrode surface.^7,51^

**Fig. 8:**
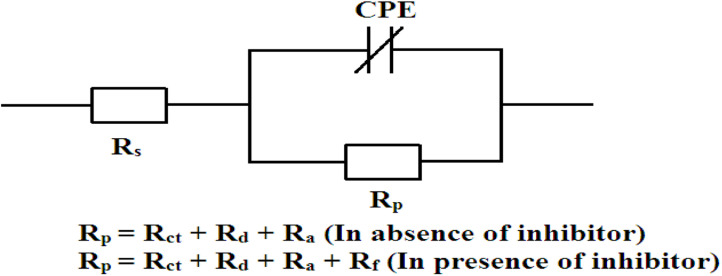
Electrical equivalent circuit model used to fit the experimental EIS data.

To account for this, a constant phase element (CPE) is incorporated into the equivalent circuit model, as depicted in Fig. 8, to accurately fit the experimental EIS data. In this circuit, the CPE is connected in parallel with *R*_p_, and this combination is in series with the solution resistance *R*_s_. The impedance of the CPE, *Z*_CPE_, is described by eqn (9):^7^

9 *Z*_CPE_ = *Q*^−1^(*iω*)^−*n*^

where, *Q* is the proportionality constant, *ω* is the angular frequency, and *n* characterizes the degree of surface heterogeneity. The CPE behaves like a capacitor (*C*), resistor (*R*), or inductor (*L*) when *n* equals 1, 0, or −1, respectively.

Furthermore, the double layer capacitance (*C*_dl_) is related to *R*_p_ and *Q* by eqn (10):^7^

10 *C*_dl_ = (*QR*_p_^1−*n*^)^1/*n*^

The fitted EIS parameters are summarized in Table 5. Results indicate that increasing the concentration of corrosion inhibitors leads to a significant increase in *R*_p_ values, while *C*_dl_ decreases correspondingly. This trend reflects enhanced surface coverage by the adsorbed inhibitor molecules on the mild steel substrate, effectively reducing the active area for corrosion; that ultimately give rise to better inhibition efficiencies. In addition, the observed decrease in *C*_dl_ values can be attributed to an increase in the thickness of the electrical double layer and/or a reduction in the local dielectric constant at the metal–solution interface.

**Table 5:** Electrochemical data obtained for mild steel exposed to 1 M HCl at 298 K in the presence of various concentrations of the corrosion inhibitors SAP4, SAP5, and SAP6

Medium	*C* (M)	*R*_s_ (Ω cm^2^)	*R*_p_ (Ω cm^2^)	*C*_dl_ (µF cm^−2^)	*n*	CPE (µF s^*n*−1^)	*η*_EIS_%
Blank	1	1.09 ± 0.4	35 ± 1.2	121	0.769 ± 0.02	427.0	—
SAP4	1 × 10^−3^	0.783 ± 0.9	349 ± 1.5	50	0.811 ± 0.09	107.5	90
	5 × 10^−4^	2.173 ± 0.5	217 ± 1.4	67	0.854 ± 0.04	123.9	84
	1 × 10^−4^	1.073 ± 0.5	153 ± 1.4	77	0.797 ± 0.03	189.4	77
	5 × 10^−5^	1.56 ± 0.2	82 ± 1.9	83	0.826 ± 0.02	198.0	57
SAP5	1 × 10^−3^	2.804 ± 0.8	497 ± 1.1	40	0.854 ± 0.10	71.5	93
	5 × 10^−4^	1.992 ± 0.2	217 ± 1.8	66	0.855 ± 0.02	123.0	84
	1 × 10^−4^	1.851 ± 0.1	112 ± 2.4	68	0.818 ± 0.08	165.4	69
	5 × 10^−5^	1.59 ± 0.2	98 ± 1.9	72	0.811 ± 0.06	183.3	64
SAP6	1 × 10^−3^	1.863 ± 0.3	240 ± 1.6	51	0.771 ± 0.05	140.0	85
	5 × 10^−4^	1.124 ± 0.1	149 ± 2.3	66	0.763 ± 0.09	149.3	77
	1 × 10^−4^	1.44 ± 0.4	100 ± 1.5	72	0.809 ± 0.12	184.6	65
	5 × 10^−5^	1.43 ± 0.2	65 ± 2.1	86	0.781 ± 0.04	268.0	46

The *R*_p_ values can further be used to estimate the surface coverage (*θ*) and the corrosion inhibition efficiency (*η*_EIS_%) provided by the applied inhibitor molecules, using eqn (11) and (12):^7,51^11
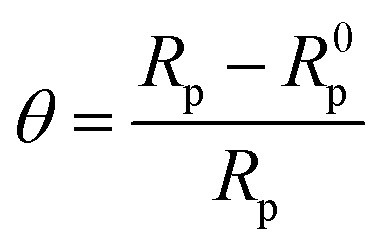
12
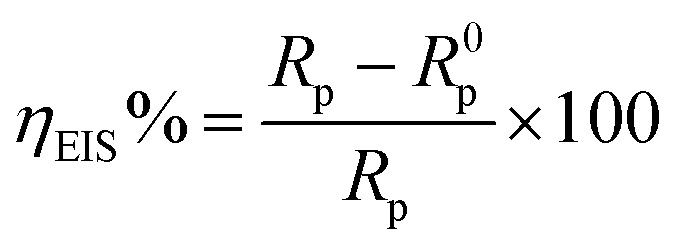
where *R*_p_ and *R*^0^_p_ represent the polarization resistance in the presence and absence of the inhibitor, respectively.

At the optimal inhibitor concentration, the maximum inhibition efficiency calculated from the polarization resistance values reached 93% for SAP5, while SAP4 and SAP6 showed 90% and 85%, respectively.

Although a slight shift in *E*_corr_ toward more positive values is observed, the magnitude of displacement is less than 85 mV compared to the blank solution. According to standard criteria, this indicates that the studied inhibitors act as mixed-type inhibitors, affecting both anodic metal dissolution and cathodic hydrogen evolution reactions, with a slight predominance toward the anodic process which act mainly by forming a protective film on the anodic surfaces, thus blocking the electrochemical reaction of metal dissolution. The observed outcomes are attributable to an increased quantity of electron-donating heteroatoms within SAP5, enhancing its capacity to adsorb across the mild steel surface. This promotes the formation of a protective layer that achieves extensive surface coverage. Moreover, the extended conjugation of π-bonds further facilitates the effective adsorption of SAP5 molecules onto the specific mild steel surface.

### 3.7. Adsorption isotherms and thermodynamic properties

The adsorption isotherm is widely used to investigate the adsorption behavior of inhibitors on a required metal surface. Inhibitor molecules adsorb on the metallic surfaces by displacing the water molecules surrounding the corroding interface. The adsorption of inhibitor molecules onto the electrode surface is a quasi-substitution process, where inhibitor molecules from the aqueous phase replace water molecules previously adsorbed on the electrode surface.^7,54^ This displacement can be represented by eqn (13):

13 Inh(sol) + *x*H_2_O(ads) ↔ Inh(ads) + *x*H_2_O(sol)

**Fig. 9:**
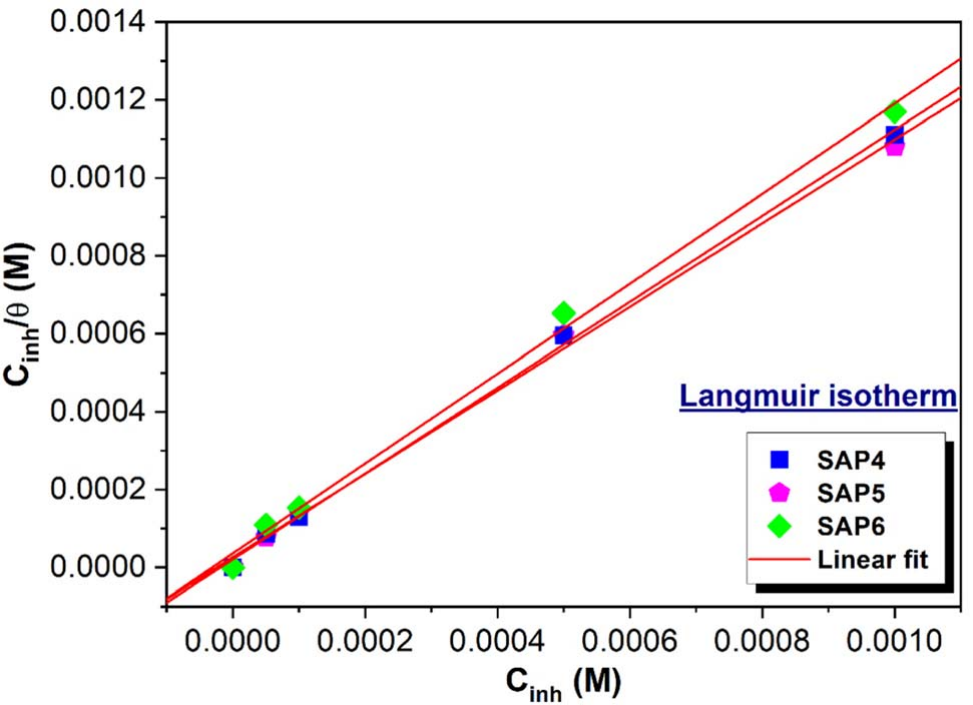
Adsorption isotherm plots of corrosion inhibitors (SAP4, SAP5 and SAP6).

In this process Inh(sol) and Inh(ads) denote the inhibitor molecules in the solution phase and those adsorbed on the electrode surface, respectively, while *x* represents the number of water molecules displaced by a single inhibitor molecule. The degree of surface coverage (*θ*), calculated from EIS measurements, along with the inhibitor concentration (*C*), has been employed to evaluate the most appropriate adsorption isotherm models. Six different adsorption isotherm models (Langmuir, Temkin, Freundlich, Frumkin, Flory–Huggins, and Al-Awady) were employed to investigate the adsorption behavior of the studied inhibitors (*vide*Fig. 9 and S1). The corresponding adsorption parameters are summarized in Tables 6 and S1. The results indicate that the adsorption of the inhibitors follows the Langmuir isotherm model, as evidenced by slope values and correlation coefficients (*R*^2^) close to unity. Eqn (14) represents the Langmuir isotherm, relating surface coverage (*θ*) to the inhibitor concentration (*C*).^4,8^

14 *C*/*θ* = 1/*K*_ads_ + *C*

where, equilibrium adsorption constant denoted by *K*_ads_. Langmuir adsorption isotherm plots gives a straight line with strong correlation coefficients (*R*^2^) values of 0.99, suggests applicability of this model.

**Table 6:** Adsorption parameters determined for corrosion inhibitors (SAP4, SAP5 and SAP6) using Langmuir adsorption isotherm

Inhibitor	Isotherm	Parameter		*R* ^2^	*K*_ads_	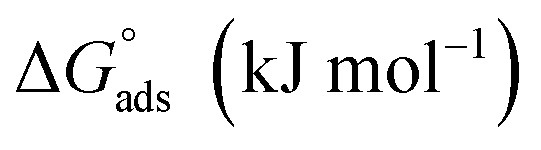
SAP4	Langmuir	Slope	1.1026	0.9993	47 472.11	−36.66
SAP5	Langmuir	Slope	1.0719	0.9986	38 217.97	−36.12
SAP6	Langmuir	Slope	1.1554	0.9982	27 649.36	−35.32

The standard Gibbs free energy change of adsorption 
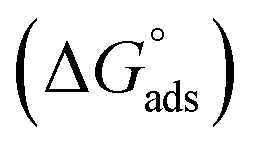
 was calculated using eqn (15):15
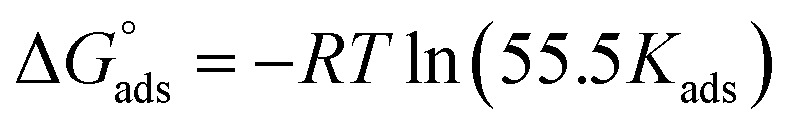
where *R* is the universal gas constant, *T* is the absolute temperature, and 55.5 represents the molar concentration of water in mol L^−1^.

It has been reported that 
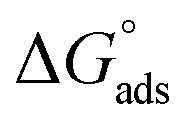
 values around −20 kJ mol^−1^ or less negative refers to the physisorption of the inhibitor on the metal surface, while 
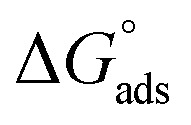
 values around −40 kJ mol^−1^ or more negative are associated with a chemisorption of the inhibitor.^55^ The adsorption behavior of the inhibitors was evaluated using the Langmuir isotherm model. The linear plots of *C*/*θ versus* C (Fig. 9) yielded correlation coefficients (*R*^2^) close to unity (0.998–0.999), confirming the applicability of the Langmuir model. The calculated adsorption equilibrium constants (*K*_ads_) were in the order of 10^4^ to 10^5^ L mol^−1^, indicating strong adsorption. Furthermore, the standard free energy of adsorption 
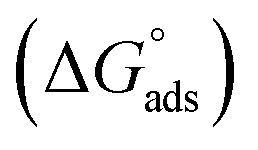
 ranged from −36.66 to −35.32 kJ mol^−1^, suggesting a mixed adsorption mechanism involving both physisorption and chemisorption.

### 3.8. Effect of temperature

**Fig. 10:**
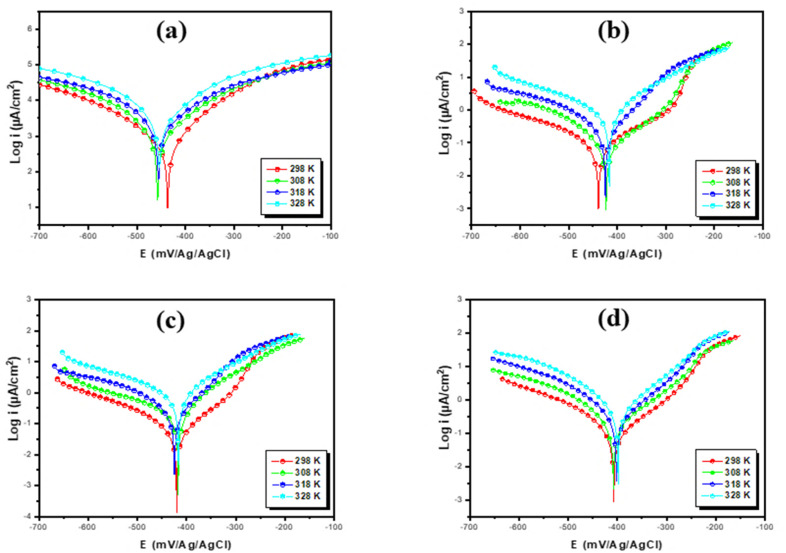
Tafel plot of mild steels exposed to (a) 1 M HCl and corrosion inhibitors (b) SAP4, (c) SAP5 and (d) SAP6 with variation of temperature (298 K to 323 K).

To assess the influence of temperature on the corrosion inhibition performance of the tested compounds, potentiodynamic polarization (PDP) measurements were conducted over a temperature range of 298–328 K. Fig. 10 presents the Tafel plots of mild steel in 1 M HCl solution before and after the addition of 1 × 10^−3^ M of each inhibitor at various temperatures. It is evident that both cathodic and anodic current densities increase with rising temperature, regardless of the presence of inhibitors. It suggests with increasing temperature the rate of corrosion increases. Although, across the temperature range of 298 K to 328 K, the inhibited systems consistently exhibit lower corrosion current densities (*i*_corr_) compared to the uninhibited ones (*vide*Table 7). It indicates investigated molecules considerably inhibited the corrosion process of mild steel also in high temperature.

**Table 7:** Temperature effect variation on potentiodynamic polarization results of mild steels in uninhibited and inhibited 1 M HCl solution

Medium	Temperature (K)	*E*_corr_ (mV)	*i*_corr_ (µA cm^−2^)	*β*_c_ (mV dec^−1^)	IE_PP_%
1 M HCl	298	437	983	150	—
	308	456	1470	136	—
	318	455	2200	116	—
	328	454	3200	115	—
SAP4	298	439	58	102	94
	308	423	129	121	91
	318	424	240	131	89
	328	416	469	133	85
SAP5	298	420	50	110	95
	308	418	128	111	91
	318	424	234	115	89
	328	417	443	102	86
SAP6	298	407	112	111	89
	308	408	205	106	86
	318	401	365	107	83
	328	397	620	113	81

### 3.9. Surface characterization

**Fig. 11:**
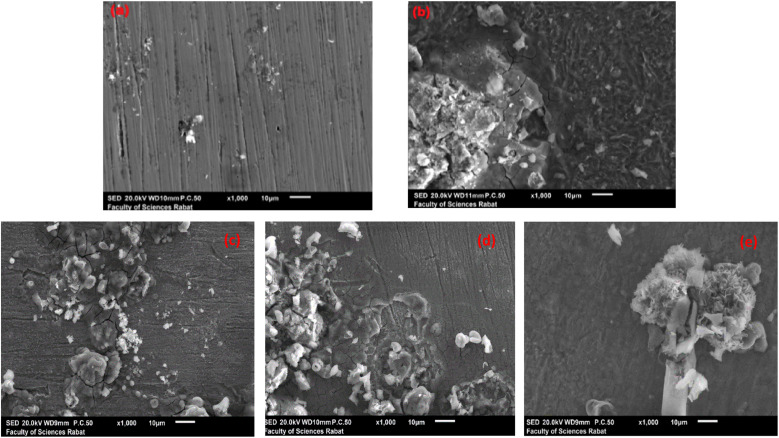
SEM micrographs of (a) polished mild steel and mild steel samples after 6 hours of immersion in (b) 1 M HCl and in 1 × 10^−3^ M solutions of the inhibitors: (c) SAP4, (d) SAP5, and (e) SAP6.

Scanning electron microscopy (SEM) images of mild steel surfaces, both without and with inhibitors in 1 M HCl solution, are shown in Fig. 11. Fig. 11(a) depicts polished metal, with scratches visible due to polishing on the metallic surface. Fig. 11(b) indicates that corrosion products are formed unevenly on the metal surface which was severely damaged by corrosion in the acidic medium. Fig. 11(c–e) present SEM images of the mild steel surface after immersion in 1 M HCl containing the optimum concentration of the tested inhibitors, which significantly reduce the corrosion reaction onto mild steel surfaces by blocking the accessible active sites. As a result, these samples exhibit reduced cracking and pitting, as the metal surface is protected by an inhibitor layer that effectively lowers the corrosion rate in the aggressive environment.

## 4. Conclusion

This study evaluated the corrosion inhibition performance of three sulfonamide-based compounds (SAP4, SAP5, and SAP6) for mild steel in 1 M HCl using combined theoretical and experimental approaches. Quantum chemical calculations (DFT) and Monte Carlo simulations indicated strong adsorption tendencies, particularly for SAP5, which exhibited the most favorable electronic properties and adsorption behavior.

Electrochemical measurements (PDP and EIS) confirmed that inhibition efficiency increases with inhibitor concentration, reaching values up to 95% at 1 × 10^−3^ M. The polarization results suggest that the inhibitors affect both anodic and cathodic reactions, indicating a predominantly mixed-type inhibition behavior.

Adsorption studies showed that the inhibitors follow the Langmuir isotherm model, with high correlation coefficients and negative 
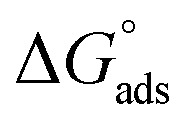
 values, suggesting spontaneous adsorption involving both physical and chemical interactions.

The inhibition performance was further evaluated over the temperature range of 298–328 K. The inhibitors maintained relatively high efficiencies at elevated temperatures, although a gradual decrease was observed with increasing temperature.

Surface analysis confirmed the formation of a protective adsorbed film on the mild steel surface, which effectively reduced corrosion. These results demonstrate that sulfonamide-based compounds, particularly SAP5, demonstrate strong potential as efficient corrosion inhibitors for mild steel in acidic environments.

## Author contributions

Conceptualization, original draft writing, reviewing, and editing: Soukaina Alaoui Mrani, Dounia Azzouni, Chahrazad El Abiad. Formal analysis, investigations, funding acquisition, reviewing, and editing: Smail Radi, Fakhreldeen Dabiellil, Mohamed Hussien. Resources, data validation, data curation, and supervision: Muhammad Shahab, Mustapha Taleb, Yousef A. Bin Jardan.

## Conflicts of interest

The authors declare that they have no competing interests.

## Supplementary Material

RA-016-D6RA02832B-s001

## Data Availability

All data generated or analyzed during this study are included in this published article. Supplementary information (SI) is available. See DOI: https://doi.org/10.1039/d6ra02832b.
