# Supplementary material for: Synergistic theoretical and electrochemical evaluation of sulfonamide-based inhibitors for mild steel corrosion in HCl
Source: RSC Adv. 2026 Jun 5;16(33):30872–88. doi: 10.1039/d6ra02832b (PMC13245320; doi:10.1039/d6ra02832b)
Supplement: RA-016-D6RA02832B-s001 [file RA-016-D6RA02832B-s001.pdf]

This journal is © The Royal Society of Chemistry 2026

Supplementary Material

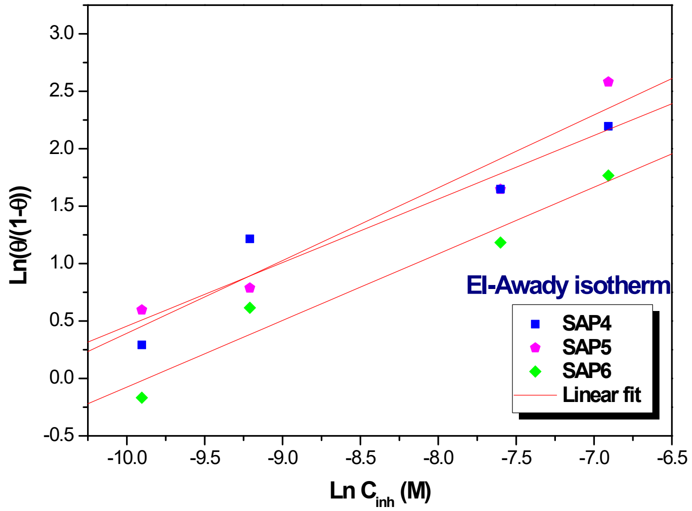

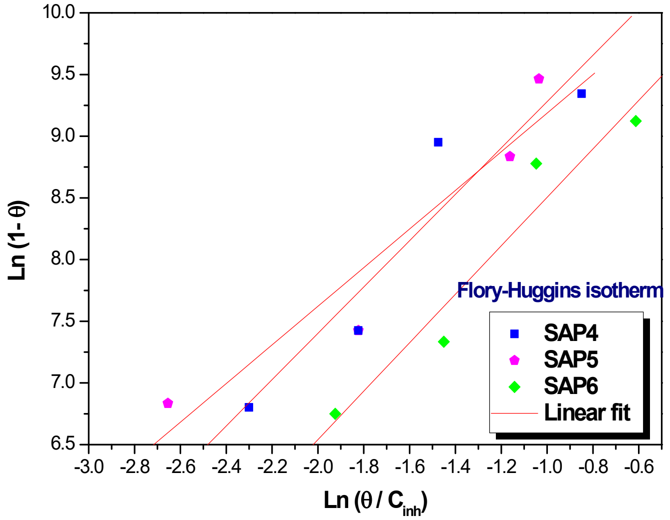

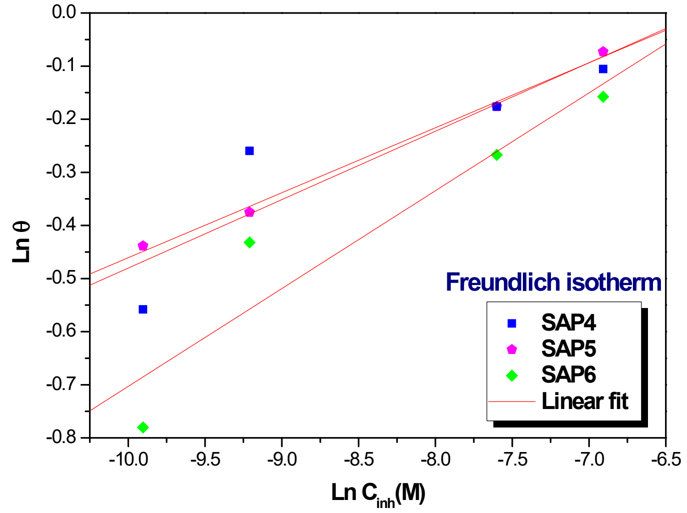

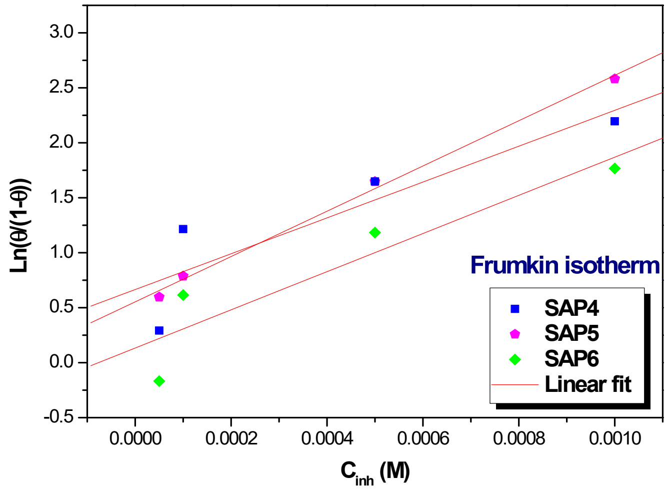

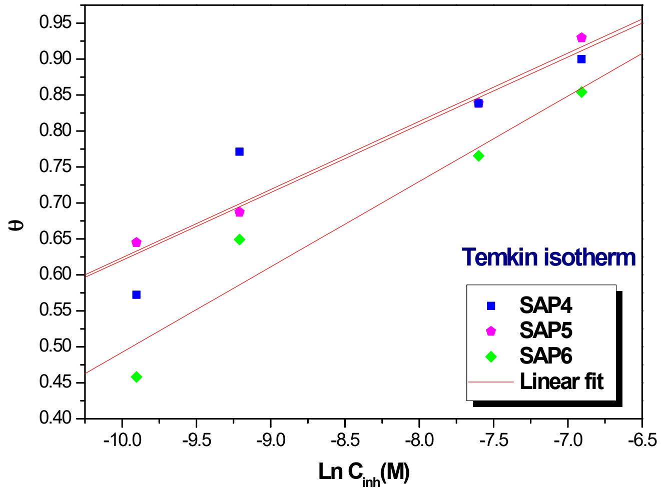

Fig. S1. Adsorption isotherm plots of corrosion inhibitors (SAP4, SAP5 and SAP6)

Table S1: Adsorption parameters determined for corrosion inhibitors (SAP4, SAP5 and SAP6) using different adsorption isotherm

Inhibitor	***Isotherm***	***Parameter***		R^2	***K***	ΔG°_{ads} ***(kJ/mol)***
SAP4	Langmuir	Slope	1.1026	0.9993	47472.11	-36.66
	Temkin	*A*	-5.3067	0.9200	15913507.53	-51.07
	Frumkin	*D*	-815.0950	0.8924	0.51	-83.10
	Freundlich	*Z*	7.7592	0.8988	2.24	-11.96
	Flory-Huggins	*X*	1.8782	0.9464	70183.60	-37.62
	El-Awady	*1/y*	1.8070	0.9550	50124.36	-36.79
SAP5	Langmuir	Slope	1.0719	0.9986	38217.97	-36.12
	Temkin	*A*	-5.2737	0.9943	15882690.53	-51.07
	Frumkin	*D*	867.8273	0.9265	1.14	-10.29

	Freundlich	*Z*	8.1686	0.9977	2.15	-11.85
	Flory-Huggins	*X*	1.5657	0.9539	46757.03	-36.62
	El-Awady	*1/y*	1.5778	0.9682	837.57	-26.65
SAP6	Langmuir	Slope	1.1554	0.9982	27649.36	-35.32
	Temkin	*A*	-4.2109	0.9639	1393220.01	-45.03
	Frumkin	*D*	867.8273	0.9265	1.14	-10.29
	Freundlich	*Z*	5.4274	0.9409	3.12	-12.78
	Flory-Huggins	*X*	1.9641	0.9685	35198.19	-35.91
	El-Awady	*1/y*	1.7238	0.9762	306.66	-24.15
